# P024. Features of headache attributed to carotid and vertebral arteries dissection

**DOI:** 10.1186/1129-2377-16-S1-A171

**Published:** 2015-09-28

**Authors:** Luca Marsili, Simone Gallerini, Manuele Bartalucci, Francesca Rossi, Sergio Pieri, Roberto Marconi

**Affiliations:** Sapienza, University of Rome, Rome, Italy; Unit of Neurology, Misericordia Hospital, Grosseto, Italy

Headache and cervical pain are common but not specific symptoms of carotid and vertebral arteries dissection. It is difficult to identify a specific pattern of pain due to dissection, useful to correctly address the diagnosis of dissection at first clinical evaluation, if other neurological signs (e.g., cranial nerves deficit, Horner's syndrome and other signs of cerebral ischemia) are not present. Recently, in the third edition of the International Classification of Headache Disorders (ICHD-III beta) diagnostic criteria for headache attributed to arterial dissection have been modified. Some Authors have suggested that this new classification is more reliable to detect carotid or vertebral arteries dissection at first clinical evaluation. Some headache features, such as, acute onset, continuous lasting and time-persistence, are currently emphasized. We have retrospectively investigated 34 patients diagnosed from January 2012 to March 2015 with cervical artery dissection. Our aim was to identify the main features of headache attributed to arterial dissection, in our cohort of patients, according to the new ICHD-III beta. We enrolled 34 patients (20 females; mean age 56 ± 11; age range 31-83), 20 of them with headache. In 10 of these 20 patients, headache was the unique symptom.

## Methods

According to ICHD-III beta, we analysed headache features in our cohort of patients. We observed that item C 3a (pain is severe and continuous for days or longer) was the most recurrent in our group of patients. The other common characteristic was the recent onset. Further studies are requested to individuate a typical clinical feature associated with headache secondary to artery dissection. We suggest that neurologists, when evaluating a recent-onset headache with a continuous and time-persistent pain, should also consider in the differential diagnosis (beyond other more common causes of secondary headache) carotid and vertebral arteries dissection.

Written informed consent to publish was obtained from the patient(s).
